# IgG4-related disease presenting with an epidural inflammatory pseudotumor: a case report

**DOI:** 10.1186/s13256-016-0838-2

**Published:** 2016-03-15

**Authors:** Nuno Ribeiro Ferreira, Rita Vaz, Sara Carmona, Sofia Mateus, Patrícia Pereira, Liliana Fernandes, Hugo Moreira, Martinha Chorão, Luís Saldanha, António Carvalho, Luís Campos

**Affiliations:** 1 Department of Internal Medicine, Hospital São Francisco Xavier, Centro Hospitalar de Lisboa Ocidental, Estrada do Forte do Alto do Duque, 1495-005 Lisboa, Portugal; 2 Department of Pathology, Hospital Egas Moniz, Centro Hospitalar de Lisboa Ocidental, Rua da Junqueira, 126, 1349-019 Lisboa, Portugal; 3 Department of Anaesthesiology, Hospital São Francisco Xavier, Centro Hospitalar de Lisboa Ocidental, Estrada do Forte do Alto do Duque, 1495-005 Lisboa, Portugal

**Keywords:** Immunoglobulin G4-related disease, Inflammatory pseudotumor

## Abstract

**Background:**

Inflammatory pseudotumor is a rare clinical condition that can be related to immunoglobulin G4 disease. Only a few cases of spinal inflammatory pseudotumors have been reported in the literature and an association with immunoglobulin G4 disease was not conclusive in any of them. We describe what we believe to be the first biopsy-proven case of an epidural inflammatory pseudotumor related to immunoglobulin G4 disease.

**Case presentation:**

A 57-year-old Caucasian woman presented to our hospital with severe paraparesis, gait disturbance, and sensory loss secondary to a relapsing epidural mass. Examination of a biopsy specimen revealed a lymphoplasmacytic infiltration with fibrosis and an immunoglobulin G4-positive plasma cell ratio of over 50 %, which are compatible with a diagnosis of immunoglobulin G4-related inflammatory pseudotumor. Our patient was successfully treated with systemic and epidural administration of glucocorticoids.

**Conclusion:**

Immunoglobulin G4-related disease is an emerging clinical condition in which central nervous system involvement is still uncommon. We describe the case of a patient with an epidural mass with medullar compression, which was proved to be an immunoglobulin G4-related epidural inflammatory pseudotumor. Our findings suggest a new manifestation of immunoglobulin G4-related disease. This disorder should be considered in the differential diagnosis of spinal tumors as a potentially treatable condition with glucocorticoids.

## Background

Immunoglobulin G4-related disease (IgG4RD) is an emerging immune-mediated condition characterized by inflammation, fibrosis, and a tendency to develop tumefactive lesions with lymphoplasmacytic infiltration rich in immunoglobulin G4 (IgG4)-positive plasma cells. It may be associated with an elevated serum IgG4 concentration, but this feature is not always present [1, 2].

Since the first case of sclerosing pancreatitis related to IgG4 was reported in 2001, several other clinical cases have emerged in the literature, aggregating in the same entity different indefinite conditions and demonstrating that this new entity may affect virtually all organs [1, 2].

In the central nervous system, IgG4RD has been described in the pituitary gland as well as in brain parenchyma and is thought to be responsible for several cases of pachymeningitis [3–6]. There have been nine cases of inflammatory pseudotumors (IPT) affecting the epidural space described in the literature but none of them were definitely considered to be related to IgG4 disease [7–14].

To the best of our knowledge, we describe the first case of an IgG4-related IPT, which was located in the epidural space and successfully treated with systemic and epidural administration of glucocorticoids.

## Case presentation

A 57-year-old Caucasian woman with a history of Graves’ disease under substitutive treatment with levothyroxine 0.1mg per day regularly attended consultations with a rheumatologist for fibromyalgia over the past 6 years.

About 2 years and 6 months prior to the current presentation, she started to complain of dorso-lumbar pain radiating to her lower back, gluteal muscles, and thighs that was partially relieved with non-steroid anti-inflammatory drugs. The pain gradually worsened and, 6 months later, she noticed numbness and weakness in both legs, associated with asthenia, extreme fatigue, and weight loss of 10 pounds in 6 months. No fever, night sweats, or urinary and fecal incontinence were reported.

**Fig. 1:**
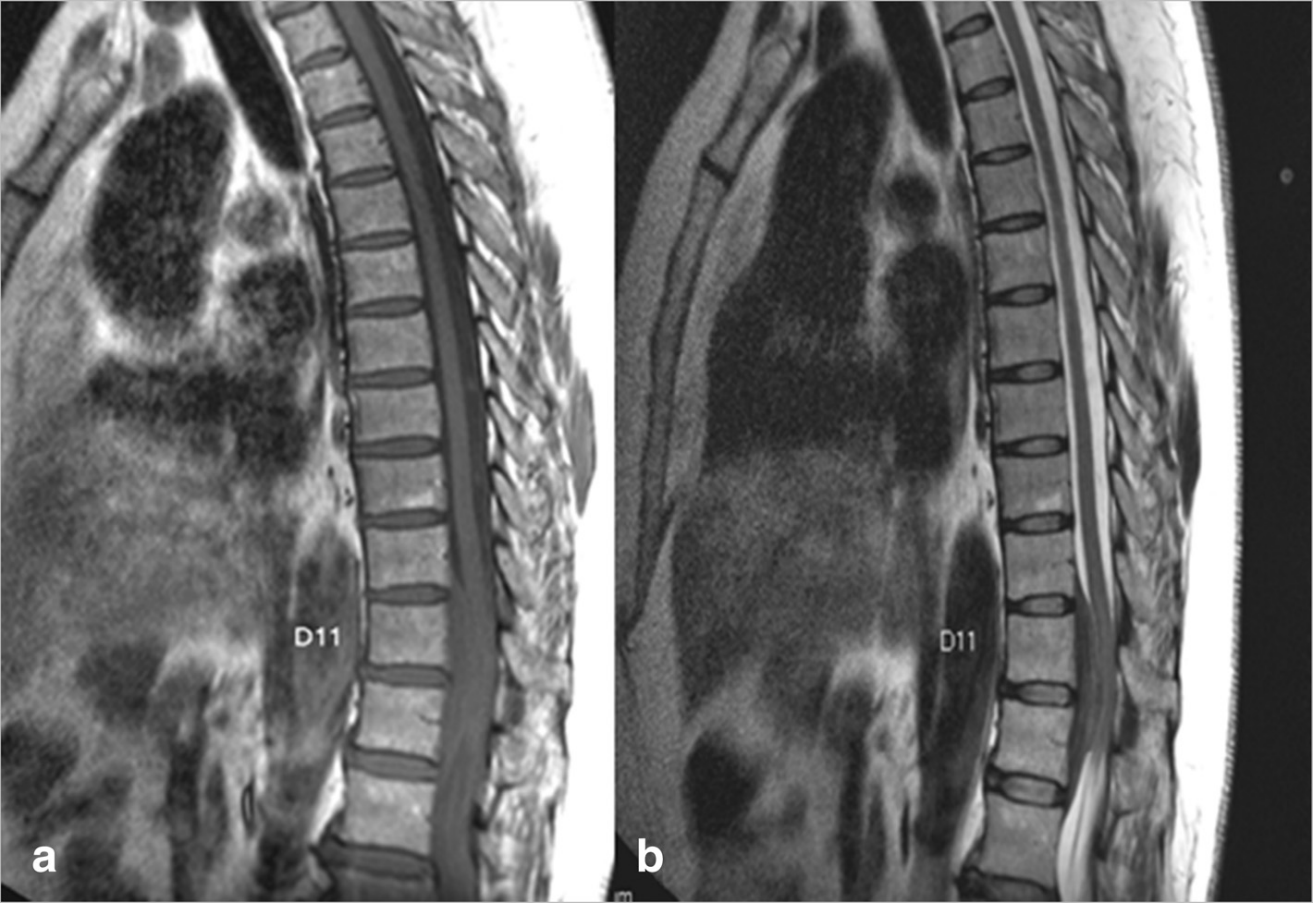
Gadolinium-enhanced magnetic resonance images showing an epidural mass at the D10–D12 level with cord compression and intense homogeneous contrast enhancement in a T1 series. **a** T1 sagittal view. **b** T2 sagittal view

**Fig. 2:**
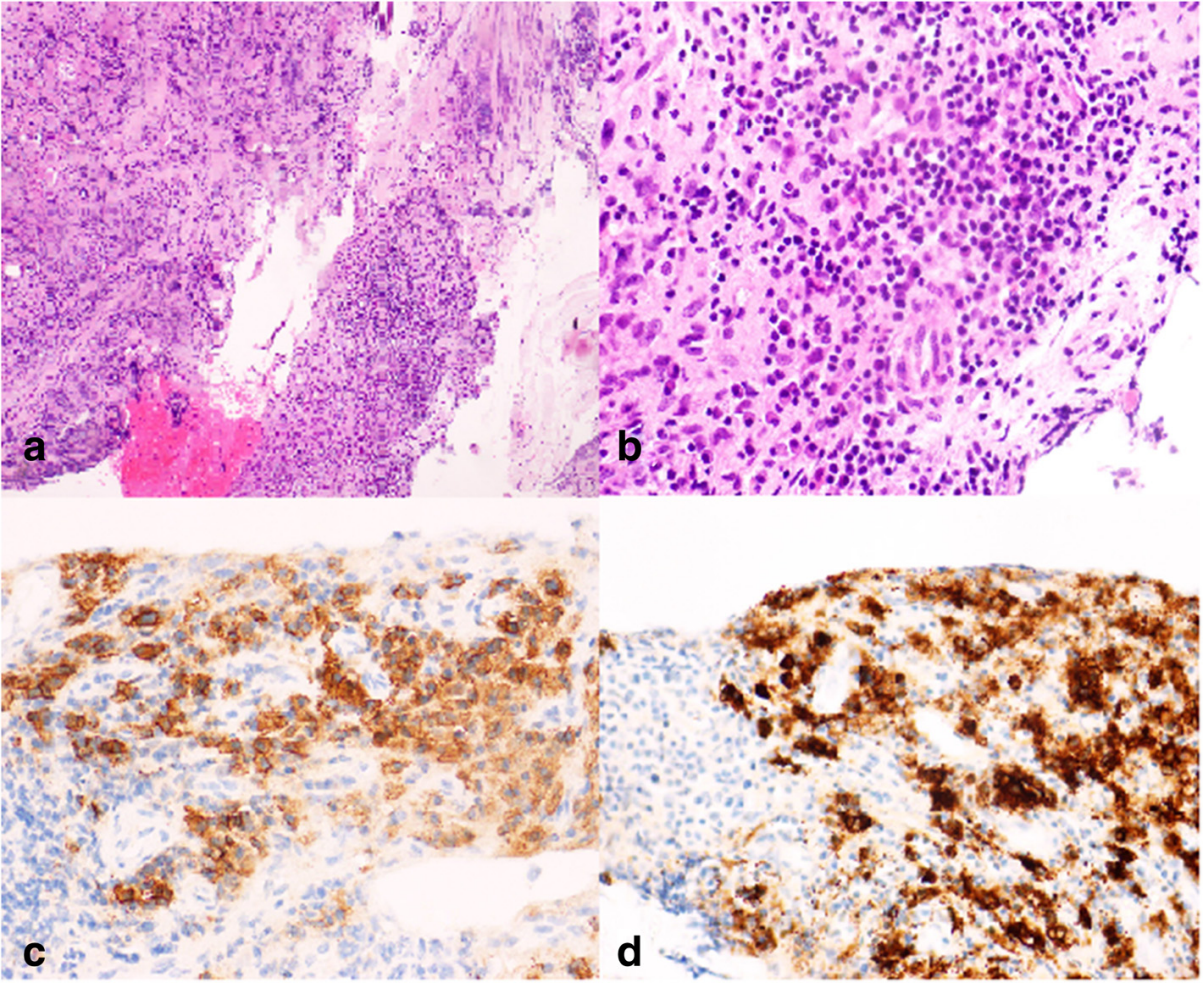
Histology of the epidural mass. **a** Chronic inflammatory lymphoplasmacytic infiltrate with fibrosis. No phlebitis was observed (hematoxylin-eosin, magnification 40×). **b** Chronic inflammatory infiltrate (hematoxylin-eosin, magnification 400×). **c** Immunohistochemistry with plasma cells (CD138^+^) in the infiltrate. **d** Immunohistochemistry with IgG4^+^ plasma cells representing more than 50 % of the total cellularity

Her physical examination revealed a grade III on the modified McCormick paraparesis scale, bilateral leg hypoesthesia, deep tendon hyper-reflexia, and bilateral Babinski signs. Her gait was unstable with no lateralization. A dorso-lumbar gadolinium-enhanced magnetic resonance image showed an expansile epidural mass from D10 to D12, which occupied all the anteroposterior diameter of her central dorsal spinal canal with cord compression and intense homogeneous contrast enhancement in a T1 series (Fig. 1a, b). Our patient was admitted to our emergency department and referred to the neurosurgery department, where she underwent a D10–D12 decompression laminectomy with subtotal resection of the mass. At this time, the presumptive diagnosis was of an epidural malignancy such as lymphoma, myeloma, or metastatic tumor. Histopathologic examination of the resected specimen, however, revealed a lymphoplasmacytic infiltration and fibrosis with no evidence of neoplastic cells (Fig. 2a, b).

Three days after surgery, our patient had no pain and she was discharged 1 week later with no neurological deficits.

**Fig. 3:**
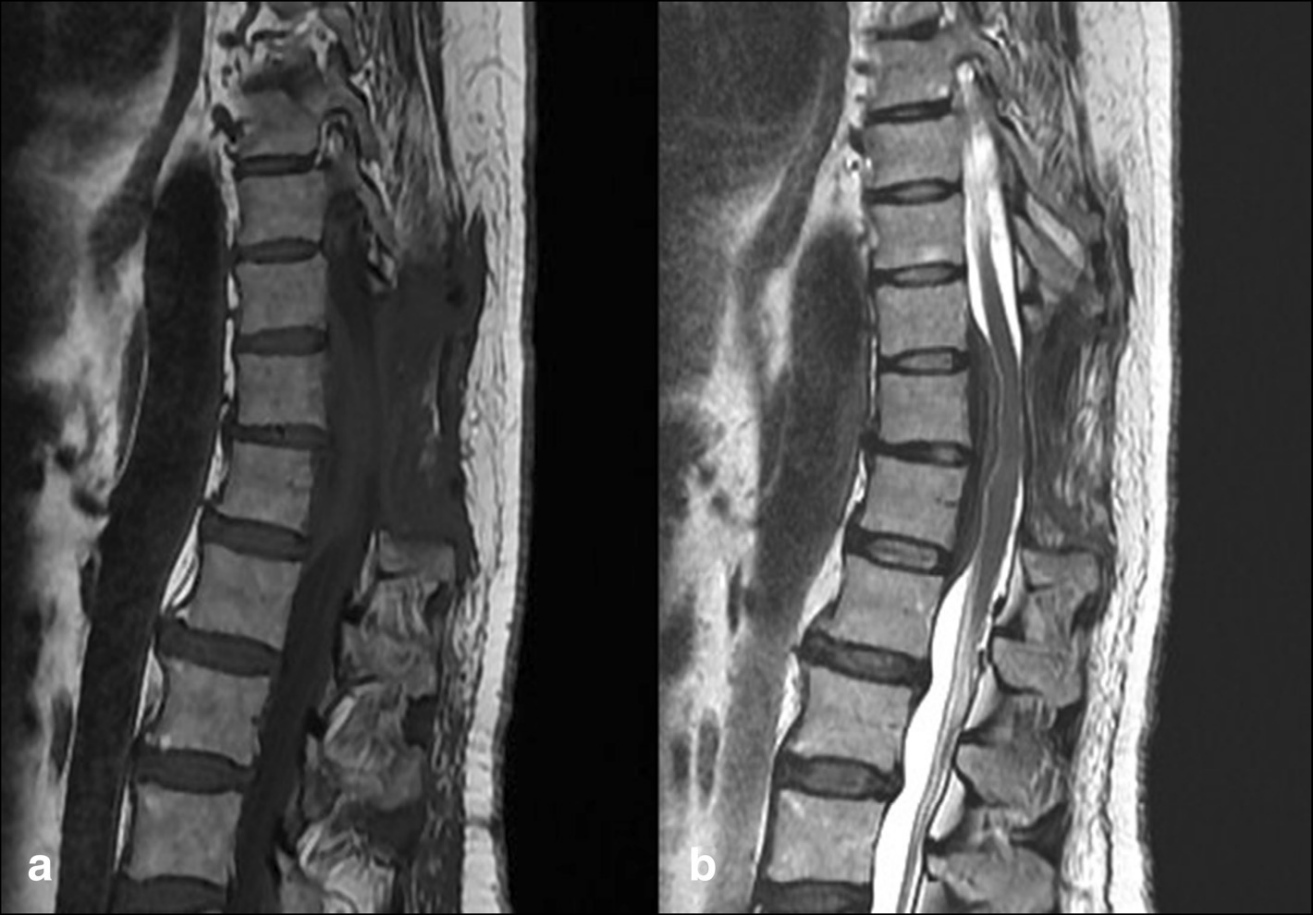
Gadolinium-enhanced magnetic resonance images 3 weeks after the first surgery showing regrowth of the epidural mass in the same localization. **a** T1 sagittal view. **b** T2 sagittal view

Three weeks after surgery, her clinical condition relapsed with dorso-lumbar radicular pain and signs of medullar compression. Magnetic resonance images showed a regrowth of the mass with the same localization and radiological characteristics (Fig. 3a, b). She underwent a second surgery and a microscopic analysis of the tumor fragment showed a mass with infiltration of lymphocytes and plasma cells with extensive fibrosis. She was discharged 2 weeks later without a definite diagnosis.

After her initial improvement, 2 months after the second surgery, she noticed a new onset of severe upper lumbar pain and progressive paraparesis, as well as asthenia and fatigue. She was admitted to our internal medicine department for further investigation. At this time, a third magnetic resonance scan revealed a D10–D12 relapsing epidural mass with persistent cord compression. A neurological examination showed sensitivity level with D10 and severe grade IV paraparesis with sensory disturbance. A third surgery was performed and the tumor was removed from her epidural space. At this time, our patient was confined to a wheelchair.

A blood analysis revealed normocytic normochromic anemia (hemoglobin 10.4g/dL) with a white cell count within the normal range. Results of a protein electrophoresis were normal. A thoracic and abdominal computerized tomography scan, as well as thyroid and breast ultrasounds, were unable to detect neoplastic disease. A bone marrow biopsy, cytometry immunophenotyping, and immunohistochemical studies revealed no cellular abnormalities. Our patient’s Ig levels revealed a mild elevation in IgG (1030mg/dL) but her levels of IgG subtypes were within normal ranges, including IgG4 (0.662g/L; normal range 0.01–2.91g/L). Her erythrocyte sedimentation rate was 24mm/h and her level of lactate dehydrogenase was normal. Blood and cerebrospinal fluid microbiological cultures were all negative, including for mycobacteria. A microscopic analysis of the mass obtained in her third surgery and the revision of previous operative specimens all showed a chronic inflammatory infiltrate and fibrosis, and a diagnosis of IPT was proposed. Our patient was treated with oral prednisolone 1mg/kg/day and epidural administration of methylprednisolone acetate (80mg/week) and ropivacaine (16mg/week) to control her severe back pain.

Her clinical condition improved over the next 6 weeks and she recovered muscular strength. *A posteriori*, IgG4 immunostaining revealed a majority of IgG4-positive plasma cells in the lesion (>50 %), confirming the diagnosis of IgG4-related epidural IPT (Fig. 2c, d).

**Fig. 4:**
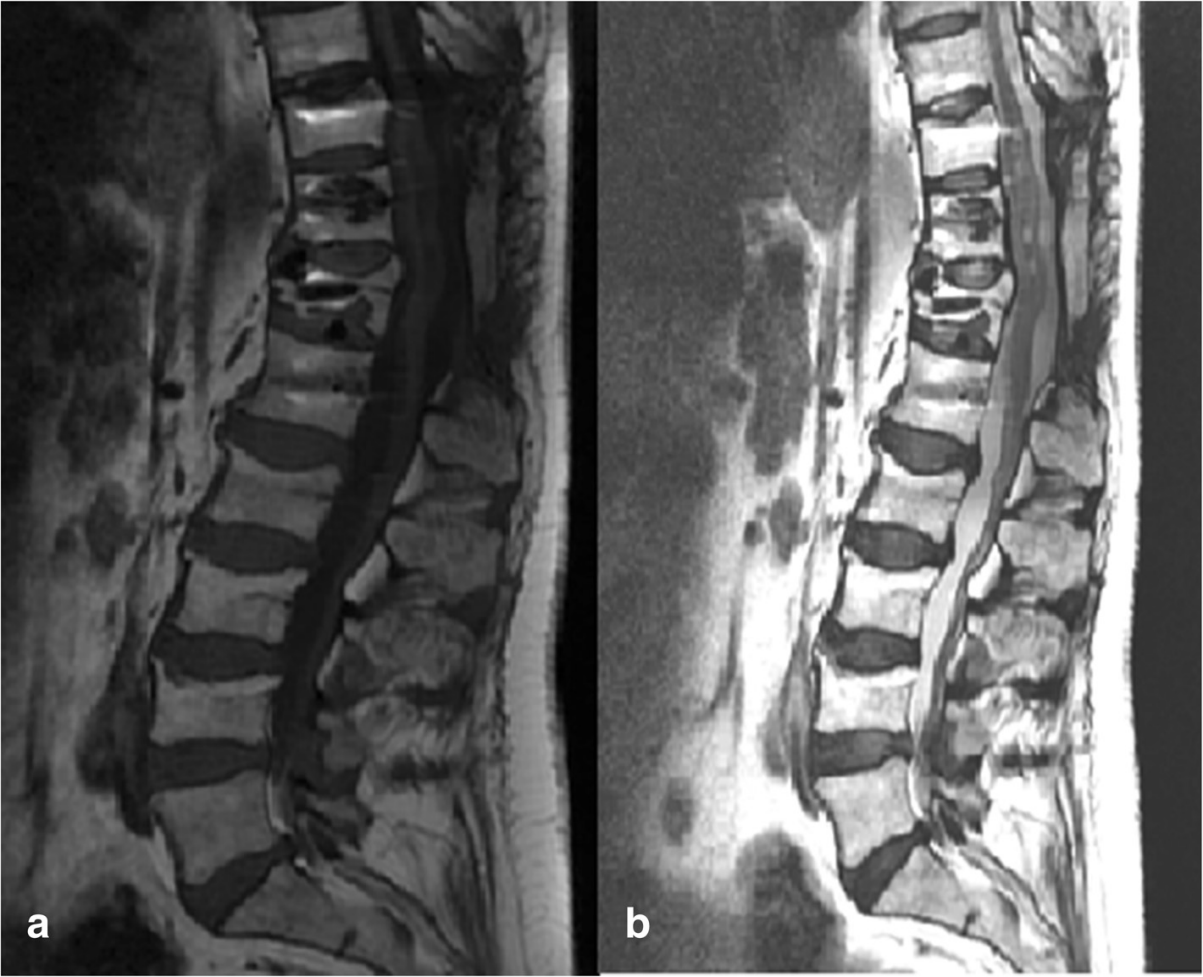
Magnetic resonance imaging 8 weeks after oral prednisolone and epidural administration of methylprednisolone acetate showing no regrowth of the mass. **a** T1 sagittal view. **b** T2 sagittal view

After 8 weeks of oral corticotherapy and weekly epidural administration of methylprednisolone, magnetic resonance imaging showed no regrowth of the mass in follow-up consultations at 3 and 6 months (Fig. 4a, b). Presently, our patient has mild hypoesthesia and severe chronic pain, probably as a result of chronic medullar compression and established epidural fibrosis.

## Discussion

IgG4RD is a recently recognized disorder that can affect any organ with multiple clinical manifestations that makes the diagnosis a challenge [1, 2].

Since IgG4RD was first recognized, an effort has been made to establish a uniform nomenclature and comprehensive diagnostic criteria. According to the diagnostic criteria suggested in a consensus statement on IgG4RD by Deshpande *et al*. [15], this disorder is defined by the presence of more than one of the major histopathological features of IgG4RD: lymphoplasmacytic infiltrate, fibrosis, and obliterative phlebitis. The presence of an increased number of IgG4-positive plasma cells and an IgG4-positive to IgG ratio above 40 % are also fundamental for a conclusive diagnosis [15]. Our case fulfills all these criteria. Her serum IgG4 concentration was not elevated, but this feature is found in up to 40 % of cases and is considered an additional finding for IgG4RD diagnosis [15].

The involvement of more than one organ system is frequently present and favors the diagnosis of IgG4RD [1, 2, 15]. Her previous diagnosis of Graves’ disease could be part of the clinical spectrum of IgG4RD disease [1, 2]. However, in our case, no histopathological confirmation was possible.

Much about IPTs etiology remains unknown but several cases have been linked to IgG4RD and, in some cases, IPT may be the form of presentation of IgG4RD [2, 5, 6]. We found nine previous cases of IPT in the epidural space reported in the literature, but none of them were proven to be related to IgG4RD [7–14]. Therefore, to the best of our knowledge, our report is the first to strongly suggest epidural IPT as a new manifestation of IgG4RD.

There are no randomized clinical trials on therapeutic options for IgG4RD; however, glucocorticoids have shown good results in clinical practice. Affected organs and disease extension will define treatment needs. Treatment should be started immediately when either organ dysfunction or pseudotumors are present [1]. In our case, as in other reports of IgG4RD with central nervous system involvement, there was an effective response to corticotherapy, even though decompression surgery was necessary [5, 6].

In this case, there was subacute onset of the major symptoms, which were secondary to progressive medullar compression by the enlarging pseudotumor. This is similar to what has previously been described in several reports of IgG4-related pachymeningitis, in which the thickening of the dura mater behaves as a compressive mass [5, 6]. Neither clinical presentation nor radiological features are distinctive in the differential diagnosis with other causes of a compressive spinal mass, thus correlation with laboratory and histopathological findings is essential to establish an accurate diagnosis [6, 14].

Therefore, even if more common conditions such as malignant tumors, infectious causes, or autoimmune diseases are ruled out, IgG4RD should be considered in the differential diagnosis of a spinal inflammatory mass in an attempt to initiate proper treatment and avoid the establishment of additional fibrosis [1, 6, 14].

## Conclusion

IgG4RD is an emerging disorder with multiple clinical manifestations. Central nervous system involvement is still a rare event. The case reported is the first to fulfill the histological and immunohistochemical diagnostic criteria for IgG4RD, suggesting epidural IPT as a new manifestation of this disorder. Thus, IgG4RD should be considered in the presence of a spinal mass after exclusion of more frequent causes.

In this case there was a clinical improvement with glucocorticoids and although randomized clinical trials on treatment options for IgG4RD are needed, we believe our results provide additional evidence of an effective response to corticotherapy.

## Consent

Written informed consent was obtained from the patient for publication of this case report and accompanying images. A copy of the written consent is available for review by the Editor-in-Chief of this journal.

## Abbreviations

Ig: immunoglobulin
IgG4RD: immunoglobulin G4-related disease
IPT: inflammatory pseudotumor
